# Review of Venetoclax in CLL, AML and Multiple Myeloma

**DOI:** 10.3390/jpm11060463

**Published:** 2021-05-24

**Authors:** Masa Lasica, Mary Ann Anderson

**Affiliations:** 1 Department of Haematology, St Vincent’s Hospital, Melbourne 3065, Australia; 2 Department of Haematology, Peter MacCallum Cancer Centre, Melbourne 3000, Australia; manderson@wehi.edu.au; 3 Department of Clinical Haematology, The Royal Melbourne Hospital, Melbourne 3000, Australia; 4 The Division of Blood Cells and Blood Cancer, The Walter and Eliza Hall Institute, Melbourne 3000, Australia

**Keywords:** BCL-2 inhibitor, venetoclax, CLL, AML, myeloma, efficacy, resistance, targeted therapy

## Abstract

Venetoclax is a highly selective and effective B-cell lymphoma-2 (BCL-2) inhibitor, which is able to reinstate the apoptotic potential of cancer cells. With its full repertoire yet to be explored, it has changed the therapeutic landscape in haematological malignancies, and most particularly chronic lymphocytic leukaemia (CLL), acute myeloid leukaemia (AML) and multiple myeloma (MM). In CLL, it has shown remarkable efficacy both as monotherapy and in combination therapy. Based on data from MURANO and CLL14 studies, fixed-duration combination therapy of venetoclax with anti-CD20 antibody is now the standard of care in numerous countries. In AML, although of limited efficacy as a single agent, venetoclax combination therapy has demonstrated encouraging outcomes including rapid, durable responses and acceptable toxicity, particularly in the older, unfit patient population. Multiple myeloma with translocation (t)(11;14) harbours high BCL-2/ myeloid cell leukaemia sequence-1 (MCL-1) and BCL-2/BCL-XL ratio and is, therefore, particularly suited for venetoclax-based therapy. Despite a wide ranging and evolving clinical role in these diseases, venetoclax treatment is not curative and, over time, clonal evolution and disease relapse appear to be the norm. While a variety of distinct resistance mechanisms have been identified, frequently emerging in a sub-clonal pattern, the full picture is yet to be characterised. Further illumination of the complex interplay of various factors is needed to pave the way for rational combination therapies aimed at circumventing resistance and improving durability of disease control. Serial molecular studies can aid in identification of new prognostically significant and/or targetable mutations.

## 1. Introduction

The highly conserved intrinsic pathway of apoptosis is tightly regulated by a balance between pro-apoptotic proteins (i.e., BAX, BAK, BIM, BID, BAD, PUMA and NOXA) and anti-apoptotic (i.e., BCL-2, BCL-XL, BFL-1/A1, BCL-W and MCL-1) proteins [1,2,3]. BAX and BAK execute apoptosis by triggering mitochondrial outer membrane permeabilisation (MOMP) and cell death via the mitochondrial pathway. Activation of these proteins is inhibited by BCL-2 homology 3 (BH-3)-only proteins including BIM, BID, PUMA, NOXA and BAD. By sequestering their pro-apoptotic counterparts, the anti-apoptotic proteins promote cell survival [4,5].

The link between overexpression of BCL-2 proteins and malignancy is well defined [4,5,6]. As a crucial survival mechanism, BCL-2 expression promotes tumourigenesis and therapy resistance by enabling cancer cells to evade apoptosis. Lymphoid malignancies frequently exhibit overexpression of BCL-2, making BCL-2 inhibitors a compelling therapeutic option.

**Figure 1:**
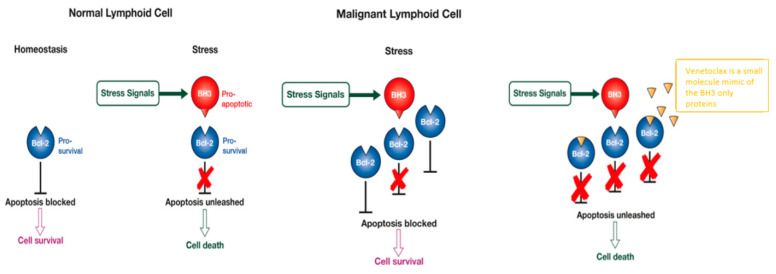
The BCL2 block on apoptosis can be overcome with venetoclax.

Venetoclax (formerly known as ABT-199) is a first-in-class, orally bioavailable, BH-3 mimetic designed by reverse engineering to produce a compound highly selective for BCL-2 with significantly lower affinity for BCL-W and BCL-XL, a molecule crucial for platelet survival [7,8,9]. Venetoclax binds to BCL-2 with high affinity, disrupting BCL-2 signaling within the cell and inducing the TP53-independent apoptotic pathway (Figure 1). It has changed the treatment paradigm of (CLL) and is of great interest in other haematological malignancies such as indolent Non-Hodgkin lymphoma (iNHL), MM and AML.

### Venetoclax Pharmacokinetics

Maximum plasma concentration is reached 5–8 h post dose and the elimination half-life (t 1/2) ranges between 17 and 41 h after a single oral dose. Bioavailability is increased by food and it is primarily metabolised via the CYP3A pathway and through the hepatic/faecal system [10]. The venetoclax area under the curve (AUC) is, therefore, 2.5 times higher in patients with severe liver disease. Co-administration with moderate or strong CYP3A inhibitors and inducers may require dose adjustment and should be avoided during dose ramp-up periods.

## 2. Venetoclax in CLL

BCL-2 is universally over-expressed in CLL cells [11], enabling them to evade apoptosis and accumulate in vivo, making CLL the ideal disease in which to test the clinical utility of venetoclax [12,13,14,15]. In view of its favourable efficacy and a tolerable toxicity profile [12,14,15], venetoclax has become standard of care for the management of both de novo and relapsed refractory CLL, demonstrating deep and durable responses regardless of adverse prognostic features such as deletion (del) (17p).

### 2.1. Venetoclax Monotherapy

The first-in-human dose-escalation study of venetoclax monotherapy in relapsed and refractory (RR) CLL was enriched for patients with a high percentage (89%) of poor clinical and/or genetic prognostic features. Despite this, the study demonstrated promising efficacy for the 400 mg dose of venetoclax with overall response rate (ORR) 79%, complete response (CR) 20%, undetectable minimal residual disease (uMRD) 15% and 15-month progression free survival (PFS) of 69%. This was the first report of uMRD among patients with relapsed and refractory CLL treated with novel agents. Unfortunately, during the dose-finding phase of the first-in-human study, three patients experienced clinical tumour lysis syndrome (TLS), including one death. Implementation of a range of TLS mitigation measures including gradual dose titration, hydration, uric acid lowering agents and protocolised monitoring significantly reduced the risk of this complication [14]. Subsequent data in a cohort of 158 patients, majority (97%) with RR CLL with del (17p), established promising tolerability and durable responses including ORR of 77%, uMRD in peripheral blood (PB) of 30% and estimated 24-month PFS of 50% [13,16].

In a rapidly evolving therapeutic landscape, the optimal sequencing of therapy for CLL remains controversial especially with a plethora of options including B-cell receptor (BCR) inhibitors such as Bruton kinase (BTK) inhibitors and Phosphatidylinositol-3 kinase (PI3K) inhibitors. Interim analysis of a Phase 2 study of venetoclax in patients with CLL whose disease had progressed after ibrutinib therapy showed durable activity with ORR of 65% at 14-months follow-up, signifying the therapeutic potential of venetoclax in this setting [17]. Similarly, a phase 2 study of 36 RR CLL patients with progressive disease during or after idelalisib therapy showed promising efficacy with ORR 67% and estimated 12-month PFS of 79% [18]. Retrospective data from 683 patients with CLL, treated with ibrutinib, idelalisib or venetoclax after initial progression on ibrutinib or idelalisib therapy, demonstrated marginally better outcomes in those treated with venetoclax (ORR 79%) versus idelalisib (ORR 46%) [19]. Further, the use of BTK inhibitors post venetoclax progression is both safe and efficacious [20]. Data from randomised, prospective trials are required to further examine sequencing strategies for these compounds.

### 2.2. Combination Therapy

Despite its effectiveness, drug resistance, toxicity, burden on the patients and compromised compliance can limit prolonged venetoclax monotherapy. Combination therapy with anti-CD20 monoclonal antibodies (mAb) and/or other small molecules in CLL has been the subject of great interest with the aim of achieving deeper and more durable responses and allowing fixed-duration therapy.

The MURANO study demonstrated superior efficacy of venetoclax-rituximab (VenR) compared with bendamustine-rituximab (BR) including higher rates of uMRD at the 9-month response assessment (62% vs. 13%). Although Grade ≥3 neutropenia was more common in the VenR arm (57.7% vs. 38.8%), the rates of febrile neutropenia (3.6% vs. 9.6%) and infections (17.5% vs. 21.8%) were lower than in the bendamustine arm. Grade ≥3 TLS occurred in 3% of patients in the VenR group [15]. Sustained PFS and overall survival (OS) benefit of VenR when compared to BR (PFS 52.6 mo vs. 17 mo and 82% vs. 61%, respectively) was recently reported in the 5-year follow-up analysis. Undetectable MRD at EOT was predictive of longer PFS and most likely underpins the superior durability of VenR. However, traditional high-risk genetic features including unmutated IGVH, del (17p) and complex karyotype continue to confer an inferior long-term outcome, carrying a higher risk of MRD conversion and relapse [21].

The CLL14 study established the venetoclax-obinutuzumab combination as a fixed-duration treatment option for patients with treatment-naïve (TN) CLL with co-morbidities including a Cumulative Illness Rating Score (CIRS) of greater than 6 or calculated creatinine clearance (CrCl) of <70 mL/min [22]. At a median follow-up of 39.6 months with all patients being off therapy for at least 2 years, the venetoclax-obinutuzumab cohort had a significantly longer PFS than the chlorambucil-obinutuzumab arm (HR 0.31, 95% CI 0.22–0.44, *p* < 0.0001). The median PFS was not reached in the former and was 35.6 months in the latter group. The most frequent Grade ≥3 adverse event (AE) was neutropenia (53%) [23]. This has made available an effective chemotherapy-free treatment option for patients with comorbidities and high-risk genetics.

Fixed-duration venetoclax-based regimes in the setting of treatment-naïve and RR CLL call attention to the efficacy of re-treatment with venetoclax in those who progress post the initial venetoclax therapy. A recent retrospective analysis of 25 patients reported encouraging results including ORR 72.2% with an estimated 12-month PFS of 69% [24]. Similarly, a small group of 11 patients, with evaluable responses in the MURANO 4-year follow-up, had ORR of 55% [25].

The phase 1/2 study investigated the combination of venetoclax with duvelisib, an oral PI3Kd inhibitor, as an all-oral, fixed-duration MRD-guided regimen in patients with Richter’s transformation and RR CLL including del (17p) (32% of patients) and TP53 mutation (45% of patients) [26]. After 12 cycles of combination therapy, those with uMRD were able to discontinue therapy while those with detectable MRD continued on venetoclax maintenance. The recommended phase 2 dose (RP2D) of venetoclax in combination with duvelisib was 400 mg. Interim analysis of 21 patients at a median number of cycles of 7.5 (range 1–22) was encouraging with ORR for the CLL/SLL group of 94% including CR 56%, PR 39% and PB uMRD 61%. The toxicity profile was manageable with neutropenia (68%) and hypocalcaemia (32%) as the most common Grade ≥3 AEs. Liver function abnormalities and diarrhoea were mild. Extended follow-up is required to ascertain long-term outcomes, particularly in the group treated with only 12 cycles of therapy.

Venetoclax has also been combined with umbralisib, a PI3kd/CK1e dual inhibitor and ublituximab, anti-CD20 mAb (U2-Ven) in RR CLL over 12 cycles with the option of continuing umbralisib monotherapy in patients with detectable MRD. Interim analysis of 19 patients who had completed 12 cycles demonstrated promising results including ORR 100%, CR 42% and uMRD 95% and 68% in PB and BM, respectively, maintained in 4 of 5 (80%) patients at 24 months. The combination was tolerable with PI3Kd-associated Grade 3 AEs (colitis and diarrhoea) occurring in 2 patients [27]. The Ven-U2 combination in TN and RR CLL will be further explored in the ULTRA-V study (NCT03801525).

Based on synergy observed in preclinical models [28], another strategy is combination of venetoclax with BTK inhibitors, which are administered as monotherapy for the first 2–3 cycles to allow mobilisation of CLL cells from the lymph nodes and reduction in tumour burden with a view to mitigating TLS risk. Venetoclax is subsequently added as fixed-duration therapy or MRD-guided treatment discontinuation. In the CAPTIVATE study, 12 months of front-line combination therapy with ibrutinib and venetoclax resulted in 58% of undetectable MRD (uMRD). These patients were subsequently randomised to either ibrutinib or placebo with similar 1-year PFS of ≥95% across the two groups making a compelling argument for fixed-duration therapy in those who achieve uMRD. The group with detectable MRD was randomised to continue venetoclax-ibrutinib combination therapy or ibrutinib monotherapy. The 30-month PFS from treatment initiation was >95% in the uMRD and detectable MRD groups [29]. Sustained responses were also observed in the CLARITY phase 2 study, which, in 50 patients with RR CLL, examined the addition of venetoclax after 2 cycles of ibrutinib [30]. At the 38-month time point (M38), 23 patients had stopped therapy at or before M38, largely (74%) due to uMRD. A further patient achieved uMRD after M38 and was able to stop therapy. Importantly, the majority (78%) of patients who achieved MRD negativity on therapy had sustained responses despite discontinuation. The 27 patients who remained on the therapy, due to persistent MRD after 12 months of combination therapy, had an encouraging ORR of 81% with MRD responses continuing to improve over time [31].

Furthermore, response-adapted addition of venetoclax to ibrutinib as consolidation was explored in patients with one or more high-risk genetic and biochemical features. The combined therapy was continued for a maximum of 2 years while uMRD at 2 consecutive time points, 6 months apart, permitted discontinuation of venetoclax. Initial results demonstrated encouraging rates of uMRD but longer-term, serial MRD analysis is required for more robust conclusions [32]. Combination of venetoclax with zanubrutinib in TN CLL patients with del (17p) will be evaluated in the SEQUOIA study (NCT03336333). Preclinical data also suggest that the combination of venetoclax with the third generation BTKi LOXO-305 may have increased efficacy in B-cell malignancies [33].

Lastly, triplet combination therapy of venetoclax with BTK inhibitors and anti-CD20 monoclonal antibodes has recently been shown to be well tolerated and to produce deep remissions in the upfront and RR setting. A phase 2 study of venetoclax was combined with ibrutinib and obinutuzumab for a total of 14 cycles in TN (*n* = 25) and RR (*n* = 25) CLL patients. Two months after completion of therapy, ORR was 84% and 88% in TN and RR patients, respectively. Twenty-eight percent of patients in both groups had achieved CR including uMRD in both blood and bone marrow. Median progression free survival was not reached at 24.2 months and 21.5 months in TN and RR groups, respectively [34]. Similarly, fixed-duration, triplet combination of venetoclax with acalabrutinib and obinutuzumab (AVO) or rituximab (AVR) in TN (*n* = 12) or RR CLL (*n* = 12) patients, respectively, demonstrated high CR/CRi rates of 50% in both cohorts, all of which achieved uMRD [35]. The AVO triplet therapy combination was also active in a phase 2 study of 44 patients with TN CLL, including 40% with TP53 mutation. The majority (78%) achieved bone marrow (BM) uMRD after 15 months of fixed-duration therapy [36]. AVO and AVR combinations both showed tolerable toxicity profiles consistent with the individual drugs.

The addition of venetoclax to zanubrutinib and obinutuzumab was explored in the BOVen study using a MRD-directed discontinuation strategy after completion of a minimum of 10 and maximum 24 cycles of therapy. At a median follow-up of 14 months (3–18), high rates of uMRD in PB (92%) and BM (84%) were observed, prompting 77% discontinuation rates at pre-specified MRD end-points. The triplet combination therapy was well tolerated with low rates of Grade ≥3 AEs (5%), most commonly neutropenia [37].

### 2.3. Resistance Mechanism

Despite its promising efficacy, the majority of patients ultimately relapse, placing emphasis on better understanding of resistance mechanisms. It has become apparent that in patients with relapsed disease, clonal heterogeneity is the norm with multiple mechanisms contributing to disease escape. The resistance mechanisms that are emerging include mutations in the BCL-2 gene conferring reduced venetoclax binding, upregulation of BCL-2 related anti apoptotic family members, alterations in the microenvironment and TP53 pathway dysfunction. Adding to the multifaceted picture is the complex interplay between co-existing modes of resistance and clonal heterogeneity.

Upregulation of anti-apoptotic proteins, particularly MCL-1 and BCL-XL, promotes cell survival and has been identified recurrently in patients with venetoclax resistance. Selection of sub-clones, which are dependent on or over-express alternate BCL-2 family members such as MCL-1 or BCL-XL, appears to be a significant resistance pathway in many cases [38,39,40,41]. Pharmacological inhibition of these proteins restores sensitivity to venetoclax in resistant cells, further emphasizing the importance of these resistance mechanisms [39,40,42]. Recent data provide insight into the hierarchy of anti-apoptotic BCL-2 members in venetoclax resistance, suggesting that BCL-XL may be more important than MCL-1 [43].

Genomic instability has been implicated as a cause for resistance. For instance, patients with a complex karyotype and TP53 dysfunction have a significantly higher risk of disease progression on venetoclax therapy [44]. Furthermore, loss of pro-apoptotic genes such as PMAIP1 and BAXA as well as genes that regulate lymphoid development (OTUD, IKZF5, NFKB1A, ID3, UB35m NF1A and EP300) appear to correlate with resistance in CLL cell lines [40]. Moreover, whole exome sequencing and methylation profiling in a group of eight pre-treated patients with venetoclax-resistant del (17p) CLL identified recurrent mutations in several cancer-related genes including TP53, NOTCH1, CDKN2A/B, BRAF, CD274, SF3B1 and BTG1 [45]. Notably, a direct causal link between the mutations and venetoclax resistance was not demonstrated, but it highlighted diverse patterns of clonal evolution.

Acquired BCL-2 mutations (G101V [46] and D103Y [47]) have recently been identified in venetoclax-resistant CLL patients. The first to be described was the G101V mutation, observed exclusively among patients on venetoclax treatment [46]. This mutation causes reduced venetoclax binding to BCL-2, conferring an outgrowth advantage and increased survival of the mutant cells over wild type cells in competition assays [46,48]. However even among the patients harbouring G101V mutations, it only accounts for a component of their resistance to venetoclax.

Additionally, recurrent subclonal amplification of 1q23 encompassing MCL-1 and PRKAB2 has been found in a number (4/6) of patients with venetoclax-resistant CLL [40]. Mitochondrial energy metabolism and “metabolic stress” pathways appear to play a role with cells enriched with genes of the AMPK pathway, such as PRKAB2, having higher survival rates on venetoclax therapy [40].

The CLL microenvironment promotes cell proliferation and survival by stimulating the transcription of anti-apoptotic genes. Furthermore, CD40L/CD40-mediated interaction between CLL and T-cells facilitates increased expression of anti-apoptotic proteins, such as BCL-XL and MCL-1 and significantly reduced sensitivity to venetoclax [49]. Importantly, pre-clinical data suggest that the sensitivity can be restored by combination with anti-CD20 antibodies and BTK inhibitors [50,51].

Finally, more traditional high-risk features such as del 17(p), TP53 mutation, NOTCH1 mutation and unmutated IGHV status; previous Bruton kinase inhibitor (BTKi) therapy, 3 or more lines of therapy,fludarabine-refractoriness and bulky lymphadenopathyalso correlate with increased risk of BCL-2 inhibitor resistant disease [44,52]. Depth of response and MRD status are increasingly recognised as a predictor of outcome in patients treated with venetoclax-based therapy [15,29,30,52].

Better understanding of resistance mechanisms is critical not only to elucidate rationally derived combinations but also for development of new agents in order to overcome resistance pathways. Next generation BCL-2 inhibitors, such as BGB-11417, have been shown in pre-clinical studies to have increased selectivity and potency compared to venetoclax, as well as activity against G101V mutated BCL-2 [53]. Furthermore, MCL-1 inhibitors, although in their infancy [54], present a promising option for combination therapy with venetoclax in order to combat overexpression of MCL1 protein in response to venetoclax. Safety of these drugs and best therapeutic strategy warrant further evaluation. ROR1 inhibitors have also shown promising efficacy [55]. Other approaches to overcome treatment resistance include tailoring therapy to the patient and their disease, combination of agents with synergistic mechanisms of action, temporal sequencing of drugs and real-time monitoring of disease response.

## 3. Venetoclax in AML

Patients with AML who are ineligible for or refractory to intensive induction chemotherapy have a poor prognosis and limited therapeutic options despite a recent expansion in the range of new agents used in this disease. Similar to myeloma cells, BCL-2 overexpression in AML is heterogeneous [56], and not always present, but there is evidence to support apoptosis dysregulation [57]. Furthermore, BH3 profiling has demonstrated BCL-2 dependence of myeloblasts, which is in line with preclinical activity of venetoclax in AML [58,59,60,61,62], highlighting it as an attractive therapeutic strategy. Combination of venetoclax and azacitidine inhibits amino acid uptake and catabolism in leukaemia stem cells, thus offering a synergistic and novel molecular mechanism to induce cell death [63,64].

### 3.1. Venetoclax Monotherapy

Efficacy and safety of venetoclax monotherapy in relapsed and refractory AML patients was first explored in a phase 2 study [65], which despite modest efficacy with an ORR of 19%, demonstrated compelling evidence that BCL-2 dependence was a predictive marker of response. Toxicity was tolerable and included nausea, diarrhoea and vomiting as the most common AEs withfebrile neutropenia and hypokalaemia as most common Grade ≥3 AEs. Tumour lysis syndrome was not recorded.

### 3.2. Venetoclax Combination Therapy

Subsequently, several studies have combined venetoclax with standard of care hypomethylating agents (HMA) or low dose cytarabine (LDAC) in unfit patients with newly diagnosed or RR disease. A phase Ib, dose escalation and expansion study evaluated venetoclax with HMA decitabine or azacitidine in previously untreated AML patients who were ineligible for intensive chemotherapy induction. The 400 mg venetoclax daily dose in the expansion cohort was administered in a three-day ramp-up and combined with either decitabine 20 mg/m^2^ day(D) 1–5 or azacitidine 75 mg/m^2^ D1–7 of 28-day cycles. The combination resulted in high rates of rapid and durable responses including CR rates of up to 74%, nearly half (45%) of which achieved uMRD (10^−3^). At a median follow-up of 15.1 months, the overall survival for all groups was 17.5 months. Most frequent Grade ≥3 AEs included haematological toxicity, febrile neutropenia and pneumonia [64,66]. This encouraging efficacy and safety data paved the way for a phase 3 study, randomizing 431 patients with previously untreated AML who were ineligible for standard induction chemotherapy to either azacitidine-venetoclax or azacitidine alone. At a median follow-up of 20.5 months, the median overall survival was longer in the combination arm (14.7 vs. 9.6 months, *p* < 0.0001). Similarly, the rates of composite complete remission were higher in the combination arm (66.4% vs. 28.3%; *p* < 0.001) across all AML genomic risk groups, including adverse cytogenetic risk, secondary AML and those with high-risk molecular mutations. The safety data were in line with known side-effect profiles of the two agents, although the combination arm had a higher incidence of febrile neutropenia (42% vs. 19%) [67].

Similarly, the longer 10-day decitabine regimen in combination with venetoclax [68] resulted in high activity as frontline therapy in AML as well as molecularly defined subsets of RR AML excluding patients with favourable-risk cytogenetics. A post-hoc analysis of the study showed that those who achieved MRD negativity at the time of morphological remission had significantly longer OS (25.1 vs. 11.6 mo), highlighting the prognostic significance of MRD negativity [69].

Promising efficacy of azacitidine combined with alternating cladribine/LDAC (CLAD/LDAC) [70] prompted the addition of venetoclax to the combination [70]. Among 48 evaluable older (age > 60 y) or unfit patients with newly diagnosed AML, the regimen resulted in deep and durable responses including CR/CRi of 94%. Importantly, the rate of MRD negativity among those in CR was 92%. At a median follow-up of 11 months, the median OS had not been reached, while the 6- and 12-month OS rates were 86% and 70%, respectively. The median survival in patients who had reached MRD negativity was significantly longer than those who had not (10.6 m vs. NR, *p* = 0.09), yet again highlighting the prognostic significance of the MRD status. The combination was well tolerated with febrile neutropenia (42%) as the most common Grade ≥3 AE. Eight-week- mortality was 6%.

The addition of venetoclax to fludarabine, cytarabine, granulocyte-colony stimulating factor and idarubicin (FLAG-IDA) in treatment-naïve and RR AML also translated into high response rates including composite complete response of 90% in newly diagnosed and 67% in RR patients. A large portion achieved MRD negativity (69%). The deep responses allowed bridging to the allogeneic stem cell transplant in more than half of the cohort (56%) including 69% and 46% of treatment-naïve and RR patients, respectively. The most common Grade ≥3 AEs included neutropenia (50%), bacteraemia (35%) and pneumonia (21%) [71].

Similarly, recent interim analysis of a Phase II study [72] combining venetoclax with CPX-352, a liposomal formulation of cytarabine and daunorubicin, revealed encouraging efficacy in RR AML (ORR 44%, CR 6%, CRi 31%), providing a viable option of bridging onto a stem cell transplant in almost all responders. Most frequent Grade ≥3 AEs included infection, nausea, pneumonia and myelosuppression.

Although venetoclax combination therapies have demonstrated promising efficacy, there is a risk of clonal evolution and disease relapse. In particular, TP53 mutations are associated with inferior response rates, shorter disease response and higher MRD positivity in newly diagnosed AML patients treated with combination of venetoclax and decitabine, highlighting the need for novel therapies in this patient group [73]. In contrast, the combination of venetoclax with LDAC or HMA in patients with NPM1 and/or IDH mutations is significantly higher and more durable, including a response rate of 93% and RLF in excess of 4 years [74].

### 3.3. Resistance

Despite its promising efficacy, clonal evolution and drug resistance limit prolonged durability of the responses in AML patients [67]. The complexity of venetoclax resistance was discussed in the CLL section with some pathways present across different malignancies and others being more cell-type dependent. Chronic exposure to venetoclax upregulates MCL-1 and BCL-XL, resulting in acquired resistance. Importantly, sensitivity can be restored by targeting these proteins, which is of substantial therapeutic interest despite lingering concerns around the tolerability of this approach [75]. Similarly, combination of venetoclax with chemotherapy agents such as daunorubicin or cytarabine can, to a degree, reverse the sequestration of BIM mediated by MCL1, therefore rendering the myeloblasts more sensitive to venetoclax [76]. Several MCL-1 inhibitors have entered clinical studies. For example, AZD5991 in combination with venetoclax or bortezomib has shown promising efficacy in pre-clinical AML and MM models, respectively [54,77]. It is currently being evaluated in a Phase I dose-finding study in patients with RR haematological malignancies (NCT03218683).

Furthermore, while certain mutations such as NPM1 and IDH are associated with high response rates and durable remissions, FLT3, RAS or TP53 confer resistance to venetoclax-based therapies [78,79,80]. Preclinical studies using CRISPR/Cas9 in AML cells have also linked mutations in BAX, TP53 and PMAIP1 genes to venetoclax resistance [80]. Sensitivity to venetoclax therapy may also depend on the maturation phase of AML blasts with reduced BCL-2 expression in more mature leukaemia cells and greater reliance on MCL-1 to mediate oxidative phosphorylation [81]. Other proposed mechanisms include alterations in the mitochondrial metabolism [82] and epigenetic changes [65,79,83]. Repeat molecular studies can aid in the identification of new prognostically significant and/or targetable mutations.

## 4. Venetoclax in Multiple Myeloma

Despite a vast array of new drugs in the treatment of multiple myeloma, it remains an incurable condition with the majority of patients inevitably relapsing. BH3-profiling has demonstrated that myeloma cells overexpress anti-apoptotic proteins in a heterogeneous manner, making their dependency on the BCL-2 survival signal rather variable. Overexpression of BCL-2 in a subset of myeloma cells with BCL-2 survival dependency, therefore, provides an attractive therapeutic target [84]. This is particularly relevant in those harbouring the translocation (11;14), found in 15–20% of myeloma patients [85,86].

### 4.1. Pre-Clinical Development

Translocation (11;14) is associated with BCL-2 overexpression and a higher BCL-2 to MCL-1 ratio, suggesting that a favourable BCL-2 family expression profile may increase susceptibility to venetoclax [87]. As demonstrated in pre-clinical data [88], myeloma cell lines and primary myeloma samples with t(11;14) exhibit high sensitivity to venetoclax. Furthermore, the modulatory effect on BCL-2 expression by other anti-myeloma therapies highlights the potential benefit of combination therapy. For instance, dexamethasone renders myeloma cells more BCL-2 dependent while bortezomib and carfilzomib stimulate expression of the MCL-1 inhibitor NOXA, therefore reducing MCL-1 mediated venetoclax resistance [89,90,91]. Recently reported preclinical data demonstrated a synergistic cytotoxic effect of venetoclax and daratumumab in myeloma cells harbouring t(11;14) with high BCL-2expression [92].

### 4.2. Clinical Data to Date

#### 4.2.1. Monotherapy

Single agent venetoclax at daily doses of 1200 mg in a heavily pre-treated cohort with a median of 5 prior lines of therapy demonstrated encouraging efficacy, particularly in the group with t(11;14) including ORR 40% and very good partial response (VGPR) or better of 27%. Biomarker analysis highlighted a strong correlation between BCL-2/BCL-2L1 and BCL-2/MCL-1 mRNA expression ratios and response rates to venetoclax [93].

#### 4.2.2. Combination Therapy

The BELLINI phase 3 study compared the combination of venetoclax or placebo with bortezomib and dexamethasone in 291 patients with 1–3 prior lines of therapy. Thirty-five patients (12%) had detectable t(11;14) and 79% had high levels of BCL-2-2 protein by immunohistochemistry. At a median follow-up of 22.7 months, the updated analysis [94] demonstrated a significant PFS (22.4 m vs. 10.4 m, HR = 0.627) and MRD negativity [10^−5^] (13% vs. 1%) advantage in the venetoclax arm. The benefit was particularly evident in the t(11;14) subgroup, where the median PFS had not been reached in the venetoclax arm (vs. 9.3 months in the placebo arm), while low BCL-2 expression by IHC was associated with inferior outcomes. The spectrum of treatment-emergent adverse events (TEAEs) in the venetoclax arm was as expected, including diarrhoea (59%), nausea (37%), constipation (35%) as well as grade 3/4 neutropenia (18%), pneumonia (17%) and thrombocytopenia (15%). Although rates of serious infections were comparable between the two arms, treatment-related mortality was significantly higher in the venetoclax arm (14 vs. 1) with overall survival in favour of the placebo group. As further defined on subgroup analysis, the favourable risk-benefit trend was, therefore, only associated with t(11;14) or high BCL-2 expression [95].

A phase 1/2 study (NCT03314181) exploring the combination of venetoclax with daratumumab and dexamethasone with and withoutbortezomib in RR MM (VenDd +/− V) is currently recruiting. The first interim analysis of 48 patients, including 24 (50%) with t(11;14) at a median time on study of 3.6 months, demonstrated a tolerable safety profile with encouraging efficacy data in the t(11;14) group, including ORR in the VenDVd and VenDd arms of 92% and 88%, respectively. Rates of very good partial response (VGPR) or better were comparable in the two arms [96].

## 5. Conclusions

Selective targeting of BCL-2 overexpression has proven to be a paradigm shifting approach to the management of several haematological malignancies—most notably, CLL and AML. For many patients, especially those with high-risk disease, venetoclax-based therapy is more effective and better tolerated than traditional chemo-immunotherapy. This promising risk-benefit profile has seen the use of venetoclax explored in a range of both haematological and non-haematological malignancies with emerging evidence in a number of additional indications. Despite this, venetoclax is not curative and treatment is inevitably hamstrung by disease relapse over time. Ongoing research is shedding light on the complex genomic and epigenomic environment that gives rise to disease resistance. Understanding these mechanisms further will help elucidate rational drug combinations to improve long-term outcomes in patients on venetoclax therapy.

## Data Availability

Not applicable.
